# Dexmedetomidine Versus Midazolam for Propofol Sparing in Procedural Sedation of Children With Leukemia: A Consecutive Case Series

**DOI:** 10.1111/aas.70107

**Published:** 2025-07-31

**Authors:** Domenica Squillaci, Karen Console, Lara Colussi, Valentina Kiren, Marco Rabusin, Gabriele Stocco, Antonella Longo, Paolo Dalena, Egidio Barbi

**Affiliations:** ^1^ University of Trieste Trieste Italy; ^2^ Institute for Maternal and Child Health IRCCS “Burlo Garofolo” Trieste Italy

**Keywords:** children, dexmedetomidine, oncology, sedation

## Abstract

**Introduction:**

Propofol is commonly used in procedural sedation in oncology due to its rapid sedative effect and favorable recovery profile. However, several preclinical and clinical studies have demonstrated a dose‐dependent neurotoxic effect of this drug. Dexmedetomidine and midazolam are potential adjuvants that, if used as premedication, could reduce the required dose of propofol. This study compares the use of dexmedetomidine and midazolam in terms of propofol dose reduction during procedural sedation in oncology patients.

**Methods:**

This one‐year retrospective study compared the outcomes of procedural sedation, in terms of propofol‐sparing, in 24 pediatric oncology patients who received midazolam (MP group, 52 procedures) or dexmedetomidine (DP group, 51 procedures) as premedication combined with propofol during bone marrow aspiration and/or lumbar puncture procedures. Data on propofol dosage, awakening time, vital parameters, and adverse events were examined.

**Results:**

Premedication with dexmedetomidine was associated with a significantly lower dose of propofol than midazolam (2.51 vs. 4.00 mg/kg, *p* < 0.001). Wake‐up times were longer in the DP group (92 vs. 65 min; *p* = 0.045). Adverse events were very rare in both groups.

**Conclusions:**

Dexmedetomidine demonstrates superior propofol‐sparing effects compared to midazolam, although it requires longer recovery times. These results support dexmedetomidine as a promising alternative in sedation protocols in pediatric oncology.

**Editorial Comment:**

This retrospectively analysis of a single center series compared procedural sedation strategies for children involving propofol after standardized intravenous premedication with dexmedetomidine or midazolam. The findings demonstrated that dexmedetomidine in those doses and in combination with propofol confirmed sedative potency and duration more than that of the chosen midazolam premedication dosing.

**Abbreviations**
ALL: acute lymphoblastic leukemia
CVC: central venous catheter
DP group: dexmedetomidine and propofol group
HR: heart rate
IQR: interquartile ranges
MP group: midazolam and propofol group
PSSS: Pediatric Sedation State Scale
SD: standard deviation

## 1 Introduction

Procedural sedation is an essential practice in the management of pain in children with oncological diseases requiring numerous diagnostic and therapeutic interventions, such as bone biopsies, bone marrow aspirations, lumbar punctures, and central venous catheter (CVC) placement [1]. Sedation represents a key strategy to prevent physical pain, minimize anxiety and psychological trauma, and facilitate the healthcare provider's ability to perform painful or invasive procedures [2].

Most procedural sedation protocols in paediatric oncology patients incorporate propofol as the primary sedative agent due to its rapid hypnotic‐sedative effect (within 30–40 s after intravenous administration), with minimal and favorable recovery time [3]. However, in recent years, several preclinical studies have shown that propofol may induce neurotoxicity, especially during critical phases of brain development, due to neuronal apoptosis in specific brain regions, including the hippocampus and cerebral cortex [4, 5]. A recent cohort study involving 212 children with acute lymphoblastic leukemia (ALL) evaluated the impact of general anesthesia on neurocognitive function and brain structure, finding that cumulative exposure to anesthetics such as propofol, along with longer anesthesia durations, was associated with slower processing speeds and cognitive impairments [6]. Another study involving 144 children aged 6 to 12 years with high‐risk B‐cell ALL confirmed these findings, demonstrating that prolonged exposure to propofol is associated with a mild but significant decline in cognitive abilities [7]. To reduce propofol exposure and its neurotoxic effects, the use of midazolam as premedication in procedures has been introduced in recent years [8]. However, the long‐term safety of midazolam is also debated, with preclinical studies demonstrating a neurotoxic risk associated with conflicting human data [9, 10]. Dexmedetomidine, a highly effective anxiolytic and sedative, has recently been shown to have neuroprotective properties in animal model studies, while there are only indirect results in humans. Although the precise mechanisms are not fully understood, it is hypothesized that hemodynamic stabilization, attenuation of inflammatory responses, and prevention of apoptosis may contribute to these benefits [11, 12].

In light of this evidence, starting in January 2024, we changed the sedation protocol for children in the Oncology Unit, introducing either dexmedetomidine or midazolam as premedication before propofol infusion.

This study aims to compare the sedative effects of midazolam and dexmedetomidine, used as premedication, to reduce the dose of propofol required in the procedural sedations of pediatric patients.

## 2 Materials and Methods

We performed a retrospective observational study, including a consecutive case series of patients with oncological diseases who were referred to the Paediatric Oncology ward of the Institute for Maternal and Child Health IRCCS Burlo Garofolo in Trieste, Italy.

### 2.1 Definition of the Protocol

As part of an Institutional quality of care improvement program, in the fall of 2023 we developed a multidisciplinary study group, creating a panel of our hospital's most experienced paediatric oncologists, sedationists, oncology nurses, and pharmacologists. This working party designed a new protocol based on premedication with either midazolam or dexmedetomidine before painful procedures related to leukaemia. The panel members were recognized authorities in the field with relevant experience in paediatric procedural sedation and leukaemia, documented by a significant research record. We conducted a review of available literature and dedicated meetings to define the drug's preparation, dosages, and infusion times. Subsequently, we performed a preliminary number of 10 procedures (five with each drug) to pragmatically test the effectiveness of the study group's proposed dosages and the awakening time, achieving a good level of sedation with both drugs.

According to the panel's decision, we did not apply the protocol when more painful procedures, such as bone marrow or liver biopsies, should be conducted and ketamine needed to be used. The propofol‐sparing effect of ketamine is well known [13]. However, the relatively high incidence of vomiting, severe agitation, and hallucinations makes this drug less attractive for patients requiring more than 20 procedures during treatment. For this reason, the panel did not consider this option.

The inclusion and exclusion criteria used for both drugs are reported below. The new protocol started in January 2024.

Before the starting of this protocol, our institute's protocol regarding procedural sedation for performing bone‐marrow needle aspiration and lumbar puncture utilized exclusively propofol as a sedative agent combined with a local anaesthetic (lidocaine and prilocaine cream, applied on the skin at the site of procedure 60 min before the beginning). In the past, according to the sedationist's judgment, propofol could be combined with premedication with midazolam to exploit the synergistic effect of the two medications, offering the availability of an antagonist drug (i.e., flumazenil) in more fragile patients and reducing the required dose of propofol.

The dosages used by the new protocol for premedication are midazolam 0.1 mg/kg infused intravenously for 2 min, followed, after a three‐minute pause, by propofol boluses, starting with a bolus of 0.5 mg/kg or a 10‐min infusion of intravenous dexmedetomidine 1 mcg/kg, followed by propofol boluses of 0.5 mg/kg.

We administered propofol intravenously, starting with a dose of 0.5 mg/kg in 2 min as induction, followed by additional boluses (0.5 to 1 mg/kg) to keep the patient sedated without the need for any physical restraint (Pediatric Sedation State Scale—PSSS ≤ 2 [13], Table S1).

The use of a local anesthetic at the procedure site remained unchanged in the new protocol. Before starting sedation, heart rate, saturation, and capnography curve monitoring was applied, which was continued after the end of the procedure until the patient was fully awake. Prior to propofol administration, all patients were given pre‐procedural oxygenation with a face mask for 3 min to achieve denitrogenation.

Knowing the average number of admissions and procedures taking place each year, including an average of more than 100 procedures, according to the panel's decision and similar studies in the literature [14], an analysis of the available data was scheduled in a one‐year time to allow for an adequate sample size.

### 2.2 Study Design

In this study, we retrospectively reviewed all consecutive procedures performed between January 2024 and December 2024, which were recorded and divided into two groups.

The first group included procedures executed employing midazolam and propofol sedation (MP group). The second group contained procedures accomplished using dexmedetomidine and propofol sedation (DP group).

The procedures analyzed involved bone marrow aspirates, lumbar punctures, or both.

Since the preparation of the two drugs is different, in a real‐life study setting, we alternated the two different premedications for eligible patients on an alternate weekly basis to facilitate nurses' preparation of the drugs, setting of the different venous lines required, and use of infusion pumps.

#### 2.2.1 Inclusion and Exclusion Criteria

We included in the analysis all the procedures performed in patients with leukemia, aged 0–17 years, who underwent bone marrow aspiration and/or lumbar puncture (with or without intrathecal drug administration, i.e., chemotherapy) under procedural sedation with propofol combined with dexmedetomidine or midazolam.

Exclusion criteria were the non‐painful procedures other than those mentioned above, performance of bone marrow or liver biopsies, use of additional drugs during sedation (e.g., ketamine) and previous adverse reactions to both drugs used for premedication. In the case of comorbidities (hypotension, hypertension, bradycardia, atrioventricular blockade) or drug use (beta‐blockers, digoxin, amlodipine) that might contraindicate the usage of dexmedetomidine, we applied only premedication with midazolam. In the case of previous adverse reactions to midazolam (paradoxical effect), we utilized only premedication with dexmedetomidine.

#### 2.2.2 Data Collection

All sedation data were routinely recorded and collected in a database from the patients' medical records. They included sex, age, weight, type of procedure, attempts made, duration of the procedure, wake‐up time, medications employed (concentration and dose pro/kg), adverse events, and vital parameters (minimum and maximum value during sedation of heart rate and oxygen saturation).

Since this study used pre‐existing, deidentified data, the Institutional Review Board deemed this study exempt. Ethical committee approval was not requested since General Authorization to Process Personal Data for Scientific Research Purposes (authorization no. 9/2014) declared that retrospective archive studies that use ID codes, preventing the data from being traced back directly to the data subject, do not need ethics approval [15]. According to the research institute's policy, the parents signed informed consent at the first visit, agreeing that “clinical data may be used for clinical research purposes, epidemiology, study of pathologies and training, with the objective of improving knowledge, care and prevention.”

In addition, as standard Institute policy, all parents signed a detailed written informed consent for all sedation procedures, after adequate explanation of the drugs used, the effects of sedation, monitoring, and possible associated risks. No parent refused the sedation protocol, and there was no drop out.

#### 2.2.3 Endpoints

The study's primary endpoint was to verify whether the use of premedication with dexmedetomidine (DP group), compared with premedication with midazolam (MP group), significantly reduced the average propofol dosage during sedation.

The secondary endpoints intended to evaluate the differences between the MP and DP groups in awakening time, basal and intra‐procedural vital parameters, and adverse events; to evaluate clinical and demographic variables associated with the propofol dose reduction between the two groups (MP and DP).

### 2.3 Statistical Analysis

Categorical variables were presented as absolute and percentage frequencies. Continuous variables were presented as means, standard deviations (SD), medians, and interquartile ranges (IQR). Differences between groups were assessed with two‐sample t‐test, chi‐squared test, or Kruskal–Wallis test when appropriate. A *p* value below 0.05 was considered statistically significant, and a 95% confidence interval was considered for all analyses. All the tests were two‐tailed.

A multivariate linear regression model was fit to associate the dose of propofol/kg administered with the available clinical variables of interest (type of associated drug, type of procedure, number of attempts), correcting for demographic variables (age, sex).

All the statistical analyses were performed using the R software version 4.3.1.

## 3 Results

### 3.1 Baseline Characteristics of the Study Population

**FIGURE 1:**
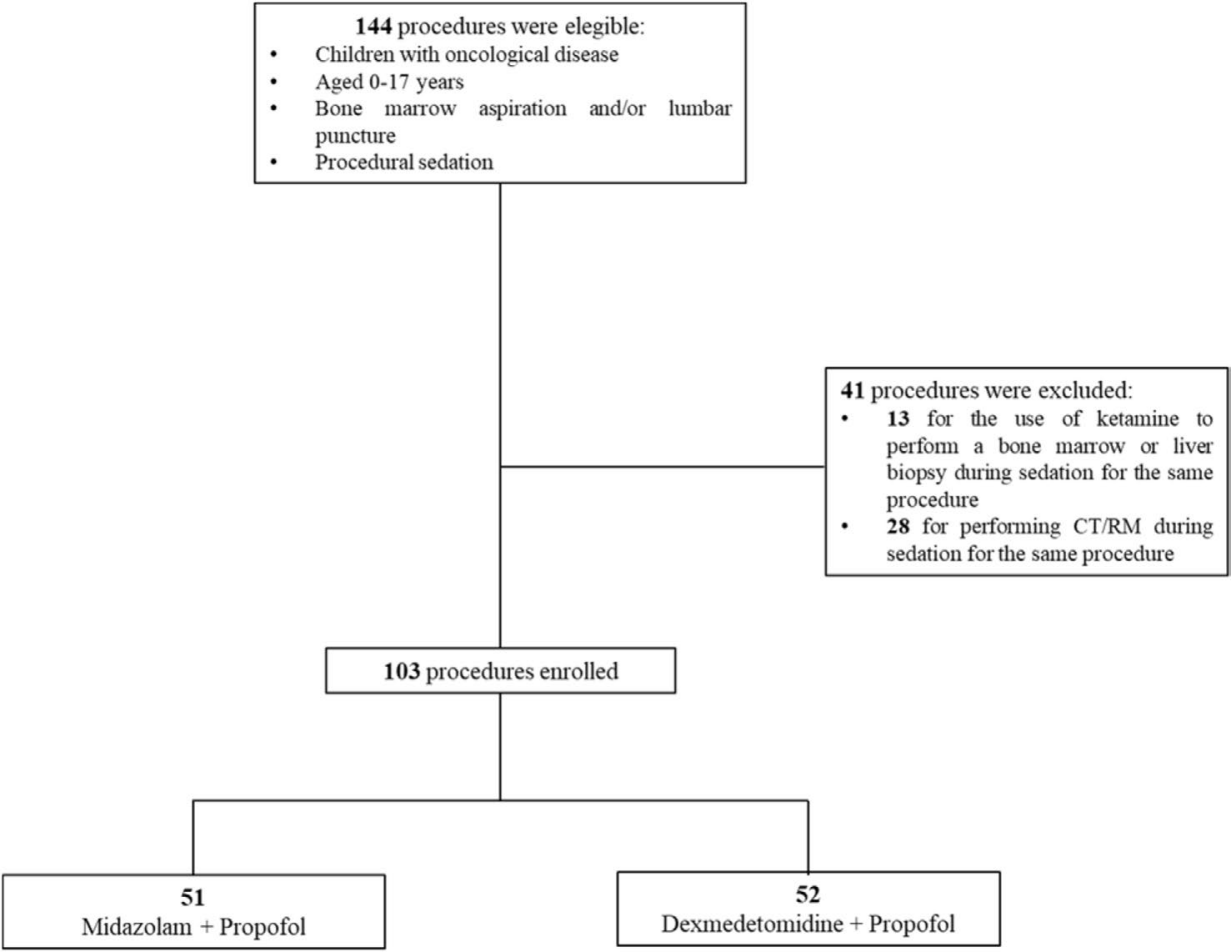
Flowchart.

From January 2024 to December 2024, 144 procedures were assessed for eligibility (Figure 1), of which 41 procedures were excluded from the study. Among the excluded procedures, we utilized ketamine in 13 cases (performance of bone marrow or liver biopsy during the procedure for bone marrow aspirate and/or lumbar puncture). In 28 cases, we performed CT or MRI during the same sedation for bone marrow aspirate and/or lumbar puncture, lengthening the sedation time and the need for additional propofol doses. We analyzed a total of 103 procedures on 24 patients. Fifty‐one procedures were premedicated with midazolam (MP group) and 52 with dexmedetomidine (DP group). Most patients were male (73.9%), and the mean age was 9.41 years (SD 4.45) (Table 1). When analyzing the baseline characteristics of the patients by dividing them into the two procedure groups (MP vs. DP), no statistically significant differences emerged (all *p* values > 0.05) (Table S2).

**TABLE 1:** Baseline characteristics of the study population.

Patients who underwent procedures with premedication (dexmedetomidine or midazolam) and propofol (*N* = 24)		
	Mean (SD)	Median (Q1; Q3)
Weight (kg)	32.06 (16.54)	29.4 (17.84; 43.10)
Age (years)	9.41 (5.45)	8.8 (4.96; 15.26)
Total number of procedures	4.48 (4.13)	4.0 (2.00; 5.00)
Lumbar puncture	3.22 (2.91)	3.0 (1.00; 4.00)
Bone marrow aspirate	2.43 (2.45)	2.0 (1.00; 3.50)

### 3.2 Main Results

**FIGURE 2:**
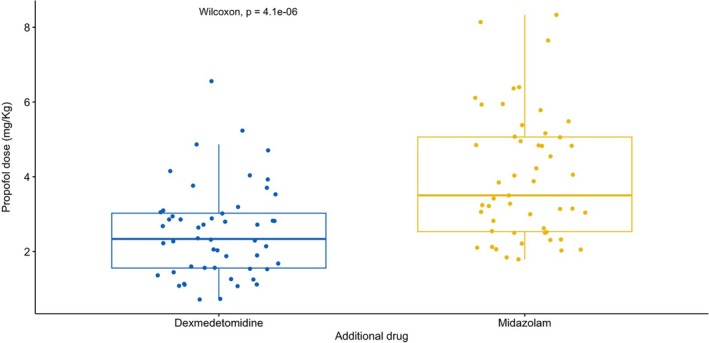
Comparison of propofol/kg dose distributions in the two groups: Dexmedetomidine and propofol vs. midazolam and propofol.

Regarding the primary endpoint, patients in the DP group required a significantly lower dose of propofol than the MP group (2.51 mg/kg vs. 4.00 mg/kg respectively, *p* < 0.001) (Table 2, Figure 2). The linear regression model also confirmed this finding when adjusting for age, gender, type of procedures done (bone marrow aspirates, lumbar punctures) and number of attempts executed (Table S3). When midazolam was given instead of dexmedetomidine, the propofol/kg administered increased on average by 1.925 mg/kg, all other variables being equal (*p* < 0.001, Table S3). If we only consider procedures involving lumbar punctures, the use of dexmedetomidine again reduced the required dose of propofol more than midazolam (mean dose of 2.23 mg/kg in the DP group vs. 3.90 mg/kg in the MP group, *p* < 0.001) (Table S4).

**TABLE 2:** Primary endpoint: Comparison of the average propofol dose/kg between the MP and DP groups.

Propofol dose (mg/kg)	DP group	MP group	*p*
Number of procedures	52	51	< 0.001
Mean (SD)	2.51 (1.22)	4.00 (1.69)	
Median (Q1; Q3)	2.33 (1.55;3.03)	3.50 (2.53;5.06)	

Abbreviations: DP group, dexmedetomidine and propofol group; MP group, midazolam and propofol group.

Wake‐up time was significantly higher in patients in the DP group (mean 89.93 min in the DP group vs. 69.33 min in the MP group, *p* = 0.045) (Table 3). Data on adverse events and intra‐procedural parameters in the two groups are shown in Table 3.

**TABLE 3:** Secondary endpoints: Comparison between the two groups MP versus DP regarding awakening time, vital parameters, and adverse events.

	DP group (52 procedures)	MP group (51 procedures)	*p*
Awakening time (min)			
Mean (SD)^a^	89.93 (52.07)	69.33 (37.35)	**0.045**
Median (Q1; Q3)^a^	92.00 (50.00, 132.00)	65.00 (45.50, 90.00)	
HR max (bpm)			
Mean (SD)	90.10 (19.37)	102.98 (18.46)	**0.001**
Median (Q1; Q3)	87.50 (73.00, 106.00)	104.00 (90.00, 114.50)	
HR min (bpm)			
Mean (SD)	75.31 (17.26)	82.02 (15.35)	**0.039**
Median (Q1; Q3)	75.50 (62.00, 88.00)	85.00 (69.50, 94.00)	
SpO_2_ max (%)			
Mean (SD)	99.94 (0.24)	99.92 (0.27)	0.680
Median (Q1; Q3)	100.00 (100.00, 100.00)	100.00 (100.00, 100.00)	
SpO_2_ min (%)			
Mean (SD)	97.17 (4.17)	97.57 (2.30)	0.552
Median (Q1; Q3)	98.00 (97.00, 99.00)	98.00 (97.00, 99.00)	
Adverse events, *n* (%)	1 (2%)	1 (2%)	—

*Note:* The *p* value assesses the differences in the distributions between the two groups. Bold indicates statistically significant values (*p* < 0.05).

Abbreviations: DP group, dexmedetomidine and propofol group; HR, heart rate; MP group, midazolam and propofol group; Q, quartile range; SD, standard deviation.

^a^ 11 missing data in DP group and 12 missing data in MP group.

One patient presented repeated sedation, apnea, and desaturation following sedation with dexmedetomidine and propofol, which occurred approximately 2 h after the end of the procedure and lasted up to 5 h afterwards. An ionic channel mutation suggesting a specific pharmacogenetic profile was found [16].

## 4 Discussion

In this study, midazolam and dexmedetomidine effectively reduced propofol dose during procedural sedation, with a statistically significant advantage of dexmedetomidine over midazolam. This study is the first one comparing midazolam and dexmedetomidine as propofol‐sparing premedication in procedural sedation in children with leukemia.

Midazolam and propofol have a synergistic effect and are commonly used to enhance sedation and reduce drug doses, but they require careful monitoring due to the risk of respiratory depression. Dexmedetomidine, an emerging sedative option, showed benefits such as preservation of respiratory drive [17], reduced propofol requirements in adult studies [18, 19], improved sedation quality, and neuroprotective effects [11, 12]. This report confirmed findings from other comparative studies, highlighting how sedative premedications could improve the pharmacological profile of sedation, allowing for more effective and safer management of pediatric procedures and reducing the use of the leading hypnotic agent [20, 21]. The relevant finding of this study was that dexmedetomidine allowed a higher reduction of propofol dose when compared to midazolam, making it a viable alternative to midazolam. Our findings added to the plausible neuroprotective effect of dexmedetomidine, suggesting that this may be the preferred option. This result was especially noteworthy in consideration of recent evidence revealing an increased risk of long‐term neurocognitive impairment associated with high doses of propofol in survivors of ALL [6, 7]. According to the study by Banerjee et al., neurocognitive impairment was correlated with a cumulative dose of propofol of 100 mg/kg (relative risk 1.40, 95% CI, 1.11–1.75) [6]. Considering the average of 25 sedation procedures that children with ALL undergo, this is equivalent to 4 mg/kg propofol per procedure. In this respect, the reduction in the propofol dose achieved by premedication with dexmedetomidine in our results becomes even more relevant, also compared to the doses per procedure in the study by Alexsander et al. (2.15 vs. 4.3–5.1 mg/kg) [7].

Our findings also confirmed and expanded data from existing studies in adults concerning the use of dexmedetomidine as premedication. Indeed, several investigations demonstrated that dexmedetomidine could significantly reduce the propofol dose required for adequate sedation, thanks to its sedative and anxiolytic properties that complemented propofol [18, 19]. In addition, compared to midazolam, dexmedetomidine has an analgesic effect of some degree that may partly explain the lower need for additional propofol doses.

Finally, we examined data from 50 procedures completed before January 2024 using only propofol (P group), conducted on 15 patients whose demographic characteristics were compared to the population of the first two groups (MP + DP group), without finding statistically significant differences (Tables S5 and S6). In our real‐life experience, patients in the P group required a significantly higher dose of propofol than the groups with premedication (DP group 2.51 mg/kg vs. MP group 4.00 mg/kg vs. P group 6.10 mg/kg, *p* < 0.001) (Figure S1).

However, some aspects must be considered in this perspective. On average, awakening times in the dexmedetomidine group were longer (mean 89.93 min in the DP group vs. 69.33 min in the MP group, *p* = 0.045), and this may affect the discharge time of children admitted to Day Hospital, causing some discomfort for families. This finding aligned with the pharmacological profile of dexmedetomidine, known for its prolonged sedative effect. Dexmedetomidine‐induced sedation was characterized by a longer duration due to its mechanism of action on α2‐adrenergic receptors, enabling a more natural and physiological sedation [22]. Although this characteristic could represent a drawback regarding overall procedure duration, it could also ensure more stable sedation, reducing the need for repeated propofol doses during the procedure. It could be advantageous in pediatric oncology patients for performing additional uncomfortable procedures after sedation, such as CVC medications, or for encouraging bed rest after lumbar punctures.

Lower mean heart rate values were recorded in the dexmedetomidine group compared to the midazolam group; this effect was predictable, as dexmedetomidine induces bradycardia. However, no patients presented with significant bradycardia requiring pharmacological intervention.

Another aspect to consider was that dexmedetomidine costs were higher than those of midazolam, which could be a problem in low‐resource settings.

In terms of safety, a major adverse event was reported in the dexmedetomidine group, with one patient experiencing delayed re‐sedation and apnea, which increased care and hospital admission. This event was unexpected since the patient had already received the same association in previous sedation uneventfully, and the safety record of the association between propofol and dexmedetomidine was good. A study by Sethi et al. indicated that the midazolam group was associated with a higher prevalence of respiratory adverse events, such as desaturations, compared to the dexmedetomidine group [23]. Indeed, midazolam, being a benzodiazepine, has depressant effects on the respiratory system, particularly when combined with other sedative agents such as propofol. Nevertheless, this study underlined how even dexmedetomidine, although generally considered less associated with respiratory depression compared to other sedatives, could enhance the sedative effect of propofol in susceptible patients, increasing the risk of respiratory depression. Thus, both combinations required careful titration and monitoring to effectively prevent and manage respiratory complications. Remarkably, a rare mutation in the 3‐UTR regulatory region of SCN9A was found in this patient, which is hypothesized to make her more susceptible to the combined effects of propofol and dexmedetomidine [16]. More data are required to confirm this finding and explain the possible impact of this genetic variant.

It should be noted that most sedations did not result in significant complications, suggesting that both premedication protocols were generally safe. They offered propofol‐sparing benefits that improved sedation management in pediatric oncologic patients.

This study has some limitations to consider. The main limits are the lack of blindness, the single center, and the relatively small sample size. In fact, a larger multicenter sample could improve the statistical power and generalizability of the conclusions, especially in terms of the safety and convenience of awakening times.

Further limitations are the study's retrospective design, the lack of a formal randomization, which may lead to potential bias in the selection of procedures to be analyzed even if a weekly shift of procedures was applied.

These factors suggest that further prospective studies, with larger samples and standardized protocols, would be necessary to confirm these results. In addition, future studies could explore the use of other drugs as premedication to reduce the dose of propofol used during procedural sedation in oncology patients.

A strength is that it was a pragmatic real‐life study. The power and structure of this type of study make it possible to investigate areas of improvement in the usual care setting, in everyday clinical practice, by evaluating procedures in unselected patients [24], thus increasing external validity.

## 5 Conclusion

In conclusion, this study compared two possible approaches to reducing propofol dosage in pediatric oncology patients. It suggests that dexmedetomidine premedication may be the more effective approach, with the advantage of having a neuroprotective effect.

Larger prospective studies are needed to confirm these results and better define the role of these two premedication options in pediatric clinical practice.

## Author Contributions

Domenica Squillaci and Egidio Barbi ideated the study. Domenica Squillaci, Karen Console, and Lara Colussi wrote the first draft of the manuscript. Karen Console and Lara Colussi collected and processed the data. Paolo Dalena processed data and performed the statistical analysis. Domenica Squillaci, Karen Console, Lara Colussi, and Egidio Barbi reviewed the literature. Egidio Barbi, Valentina Kiren, Marco Rabusin, Gabriele Stocco, and Antonella Longo critically revised the manuscript for relevant intellectual content. All authors had full access to all the data in the study and had final responsibility for the decision to submit for publication. All authors read and approved the final manuscript.

## Ethics Statement

Since this study used pre‐existing, deidentified data, the Institutional Review Board deemed this study exempt. Ethical committee approval was not requested since General Authorization to Process Personal Data for Scientific Research Purposes (authorization no. 9/2014) declared that retrospective archive studies that use ID codes, preventing the data from being traced back directly to the data subject, do not need ethics approval.

## Consent

According to the research institute's policy, the parents signed informed consent at the first visit, agreeing that “clinical data may be used for clinical research purposes, epidemiology, study of pathologies and training, with the objective of improving knowledge, care and prevention.” All parents signed a written informed consent for the sedation procedures.

## Conflicts of Interest

The authors declare no conflicts of interest.

## Supporting information


**Data S1:** Supporting information.

## Data Availability

The data that support the findings of this study are available on request from the corresponding author. The data are not publicly available due to privacy or ethical restrictions.
