# Supplementary material for: Dexmedetomidine Versus Midazolam for Propofol Sparing in Procedural Sedation of Children With Leukemia: A Consecutive Case Series
Source: Acta Anaesthesiol Scand. 2025 Jul 31;69(8):e70107. doi: 10.1111/aas.70107 (PMC12311750; doi:10.1111/aas.70107)
Supplement: Supplementary file 1 — Data S1: Supporting information. [file AAS-69-0-s001.docx]

**Supplementary materials**

**Supplementary Table S1: Pediatric Sedation State Scale (PSSS)**

| **State** | **Behavior** |
| --- | --- |
| 5 | Patient is moving (purposefully or nonpurposefully) in a manner that impedes the proceduralist and requires forceful immobilization. This includes crying or shouting during the procedure, but vocalization is not required. Score is based on movement. |
| 4 | Moving during the procedure (awake or sedated) that requires gentle immobilization for positioning. May verbalize some discomfort or stress, but there is no crying or shouting that expresses stress or objection. |
| 3 | Expression of pain or anxiety on face (may verbalize discomfort), but not moving or impeding completion of the procedure. May require help positioning (as with a lumbar puncture) but does not require restraint to stop movement during the procedure. |
| 2 | Quiet (asleep or awake), not moving during procedure, and no frown (or brow furrow) indicating pain or anxiety. No verbalization of any complaint. |
| 1 | Deeply asleep with normal vital signs, but requiring airway intervention and/or assistance (eg, central or obstructive apnea, etc). |
| 0 | Sedation associated with abnormal physiologic parameters that require acute intervention (ie, oxygen saturation <90%, blood pressure is 30% lower than baseline, bradycardia receiving therapy) |

*Cravero JP, Askins N, Sriswasdi P, Tsze DS, Zurakowski D, Sinnott S. Validation of the Pediatric Sedation State Scale. Pediatrics. 2017 May;139(5):e20162897. doi: 10.1542/peds.2016-2897. PMID: 28557732; PMCID: PMC5404726.*

**Supplementary Table S2: Baseline characteristics of the population considering the two procedure groups (MP vs DP groups).**

|  | **DP group (52 procedures)** | **MP group (51 procedures)** | **p-value** |
| --- | --- | --- | --- |
| *Sex (male), n (%)* | 37 (71.2%) | 44 (86.3%) | 0.091 |
| *Age in years*  *Mean (SD)* | 7.95 (4.96) | 9.51 (5.21) | 0.123* |
| *Median (IQR)* | 7.53 (4.04-10.44) | 10.29 (4.33-11.35) |  |
| *Weight (kg),*  *Mean (SD)*  *Median (IQR)* | 28.19 (15.78)  *26.95 (17-32.10)* | 33.42 (20.32)  29.40 (17.90-35.75) | 0.148* |
| ***Type of procedure*** | | | |
| *Lumbar puncture, n (%)* | 33 (63.5%) | 41 (80.4%) | 0.079 |
| *Bone marrow aspirate, n (%)* | 29 (55.8%) | 27 (52.9%) | 0.844 |
| **The p-value assesses the differences in the distributions between the two groups.*  *DP group - dexmedetomidine and propofol group*  *MP group - midazolam and propofol group*  *IQR - interquartile range*  *SD – Standard deviation* | | | |

**Supplementary Table S3: Linear regression: associations between the dose of propofol/kg** **and the variables of interest.**

|  | **β coefficient** | **Std.error** | **p-value** |
| --- | --- | --- | --- |
| *(Intercept)* | 2.711 | 0.528 | < 0.001 |
| *Age* | -0.092 | 0.028 | 0.001 |
| *Sex (male)* | -0.142 | 0.347 | 0.682 |
| *DP/MP (MP)* | 1.925 | 0.289 | < 0.001 |
| *Num. attempts (2)* | 1.079 | 0.483 | 0.028 |
| *Num. attempts (3)* | 0.499 | 0.627 | 0.429 |
| *Num. attempts (4)* | 2.423 | 1.380 | 0.083 |
| *Num. attempts (5)* | 0.501 | 1.415 | 0.724 |
| *Lumbar puncture (yes)* | -0.132 | 0.383 | 0.731 |
| *Bone marrow aspirate (yes)* | 0.918 | 0.343 | 0.009 |

**Supplementary Table S4: Dose of propofol/kg in the two groups (MP and DP groups) regarding lumbar punctures.**

|  | **DP group (52 procedures)** | **MP group (51 procedures)** | **p-value** |
| --- | --- | --- | --- |
| *Lumbar puncture, n (%)* | 33 (63.5%) | 41 (80.4%) | 0.079 |
| *Dose propofol/kg*  *Mean (SD)*  *Median (Q1; Q3)* | 2.23 (1.06)  2.05 (1.44; 2.82) | 3.90 (1.63)  3.42 (2.52;5.06) | ***<0.001*** |

*DP group - dexmedetomidine and propofol group*

*MP group - midazolam and propofol group*

*IQR - interquartile ranges*

*SD – Standard deviation*

**Supplementary Table S5: Baseline characteristics of patients who underwent procedures with propofol alone (P group, N=15)**

|  | **Mean (SD)** | **Median (Q1;Q3)** |
| --- | --- | --- |
| ***Average age (years)*** | 8.81 (4.73) | 8.1 (4.56; 11.61) |
| ***Total number of procedures*** | 3.33 (3.20) | 1.0 (1.00; 5.50) |
| ***Lumbar puncture*** | 2.33 (2.44) | 1.0 (0.50; 4.50) |
| ***Bone marrow aspirate*** | 1.07 (1.10) | 1.0 (0.00; 2.00) |

**Supplementary Table S6: Comparison of demographic characteristics between the group of patients receiving premedication and the group using propofol alone.**

|  | **DP+MP group (24 patients)** | **P group (15 patients)** | **p-value** |
| --- | --- | --- | --- |
| Sex (male), n (%) | 17 (73.9%) | 10 (66.7%) | 0.722 |
| Age in years,  Mean (SD) | 9.41 (5.45) | 8.81 (4.73) | 0.721* |
| Median (IQR) | 8.8 (4.96-15.26) | 8.1 (4.56-11.61) |  |
| Min, max | 1.12, 17.53 | 3.39, 18.11 |  |
| **The p-value assesses the differences in the distributions between the two groups.*  *DP group - dexmedetomidine and propofol group*  *MP group - midazolam and propofol group*  *IQR - interquartile ranges*  *SD – Standard deviation* | | | |

**Supplementary Figure 1: Comparison of propofol/kg dose distributions in the three groups: dexmedetomidine and propofol (DP group), midazolam and propofol (MP group) and propofol alone (P group).**

***
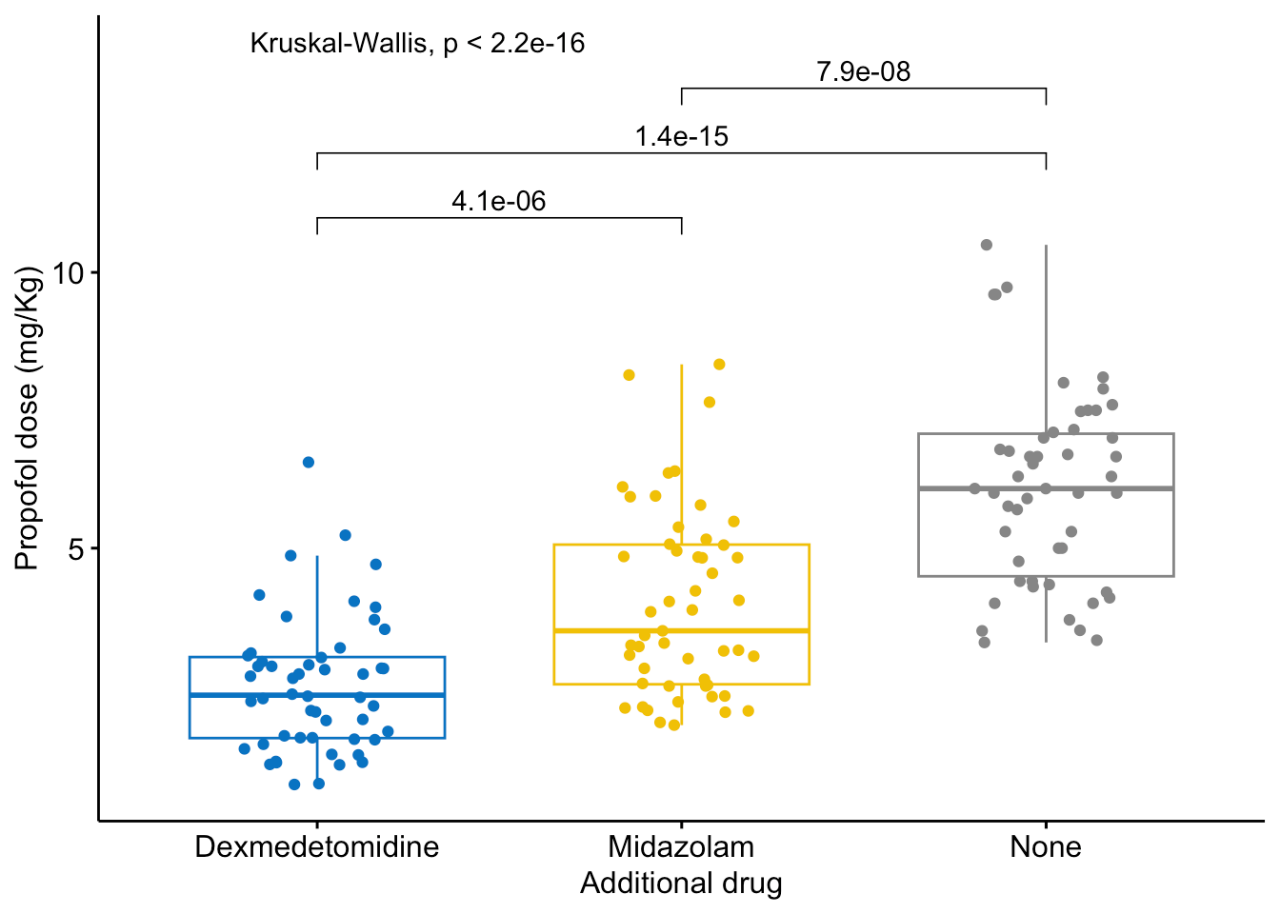
***

| Propofol dose (mg/kg) | DP group | MP group | P group |
| --- | --- | --- | --- |
| Number of procedures | 52 | 51 | 50 |
| Mean (SD) | 2.51 (1.22) | 4.00 (1.69) | 6.10 (1.76) |
| Median (Q1; Q3) | 2.33 (1.55;3.03) | 3.50 (2.53;5.06) | 6.08 (4.40;7.10) |

The figure shows the p-values of the three pairwise comparisons between the three groups and of their overall comparison.
